# High efficiency generalized transduction in *Escherichia coli* O157:H7

**DOI:** 10.12688/f1000research.2-7.v1

**Published:** 2013-01-10

**Authors:** Martin G Marinus, Anthony R Poteete

**Affiliations:** 1 Biochemistry and Molecular Pharmacology, University of Massachusetts Medical School, Worcester MA, 01605, USA; 2 Department of Microbiology and Physiological Systems, University of Massachusetts Medical School, Worcester, MA 01605, USA

## Abstract

Genetic manipulation in enterohemorrhagic *E. coli* O157:H7 is currently restricted to recombineering, a method that utilizes the recombination system of bacteriophage lambda, to introduce gene replacements and base changes *inter alia* into the genome. Bacteriophage 933W is a prophage in *E. coli *O157:H7 strain EDL933, which encodes the genes ( *stx2AB*) for the production of Shiga toxin which is the basis for the potentially fatal Hemolytic Uremic Syndrome in infected humans. We replaced the *stx2AB* genes with a kanamycin cassette using recombineering. After induction of the prophage by ultra-violet light, we found that bacteriophage lysates were capable of transducing to wildtype, point mutations in the lactose, arabinose and maltose genes. The lysates could also transduce tetracycline resistant cassettes. Bacteriophage 933W is also efficient at transducing markers in *E. coli* K-12. Co-transduction experiments indicated that the maximal amount of transferred DNA was likely the size of the bacteriophage genome, 61 kB. All tested transductants, in both *E. coli* K-12 and O157:H7, were kanamycin-sensitive indicating that the transducing particles contained host DNA.

## Introduction

Enterohemorrhagic *Escherichia coli* (EHEC) serotype O157:H7 is among the leading causes of food- and water-borne illnesses affecting humans in the U.S., Europe, and Japan (for review, see Donnenberg and Whittam (2001) ^1^, Kaper *et al.* (2004) ^2^ and Spears *et al.* (2006) ^3^). These bacteria are highly infectious and ingestion of only 100–200 organisms is sufficient to trigger debilitating diarrheal disease. Although most EHEC infections resolve spontaneously after 5–10 days of abdominal cramping and bloody diarrhea, approximately 2–7% of cases progress to the potentially fatal hemolytic uremic syndrome due, in part, to the production of cytotoxic Shiga toxins which are capable of promoting kidney failure (Donnenberg and Whittam (2001) ^1^, Kaper *et al.* (2004) ^2^ and Spears *et al.* (2006) ^3^). The genes for Shiga toxin ( *stx2AB*) are located on a prophage in the late region between genes Q and S and are transcribed with the late region genes from promoter P _R_’ (Tyler *et al.* (2004) ^4^).

The most widely used genetic manipulation in EHEC is recombineering, a method that utilizes the recombination system of bacteriophage lambda, to introduce gene replacements and base changes *inter alia* into the genome (Murphy (1998) ^5^, Court *et al.* (2002) ^6^). A method for using the generalized transducing bacteriophage P1 in EHEC has been reported previously but it requires the construction of galactose mutant derivatives of the recipient strain (Ho and Waldor (2007) ^7^).

In EHEC strain EDL933, the *stx2AB* genes are located on prophage 933W, the genome size of which is 61 kB (Penna *et al.* (2001) ^8^). The DNA sequence of both the prophage and free phage are known. During sequencing of the free phage genome, it was noticed that the ends of the genome could not be sequenced; the investigators interpreted this and other observations as indicating that the packaged chromosome might be terminally redundant (Plunkett *et al.* (2001) ^9^). Because terminal redundancy is a shared feature among generalized transducing phages, this finding suggested that bacteriophage 933W might be capable of packaging host DNA and transducing genetic markers. We report here that bacteriophage 933W is capable of transducing genetic markers in unmodified EHEC and *E. coli* K-12 strains.

## Methods

### Bacterial strains

The EHEC strain used principally in this paper is KM80 (Carone *et al.* unpublished document), a derivative of EDL933 which lacks prophage 933W (obtained from Dr. K.C. Murphy). Derivatives of KM80 unable to utilize lactose (GM9289), arabinose (GM9290) or maltose (GM9291) were obtained after a 20 min exposure of a log phase culture in L broth to 40 µl ethyl methane sulfonate (Sigma-Aldrich) per ml at 37°C followed by a 1/100 dilution into broth. Aliquots of the saturated culture were plated on MacConkey Agar Base (Difco) containing lactose, arabinose or maltose to detect non-fermenting colonies. The *E. coli* K-12 strains used were AB1157 ( *thr-1 araC14 leuB6*(Am)Δ *(gpt-proA)62 lacY1 tsx-33 supE44*(AS) *galK2*(Oc) *hisG4*(Oc) *rfbD1 mgl-51 rpoS396*(Am) *rpsL31*(Str ^R^) *kdgK51 xylA5 mtl-1 argE3*(Oc) *thi-1*) (Dewitt and Adelberg (1962) ^10^) (obtained from Dr. E.A. Adelberg), MM294 ( *endA1 hsdR17 supE44*(AS) *rfbD1 spoT1 thi-1*) (Meselson and Yuan (1968) ^11^) (obtained from Dr. M. Meselson), GM1731 ( *cysG98*::Tn *5*) (Shaw and Berg (1979) ^12^) and GM1748 ( *leu*::Tn *10*) (Kleckner *et al.* (1975) ^13^). (Am=amber, AS=amber suppressor, Oc=ochre). Strains containing the Tn *5* and Tn *10* mutations were obtained from Drs. C. Berg and N. Kleckner respectively. The composition of L broth and minimal medium has been described previously (Nowosielska and Marinus (2008) ^14^).

### Bacteriophage construction, induction and transduction

Replacement of the *stx2AB* genes in EDL933 by recombineering as described by Murphy and Campellone (2003) ^15^ was carried out to yield strain GM9251. In a polymerase chain reaction containing Herculase II DNA polymerase Agilent Technologies), supplied buffer, 2 µl GM1731, and primers. ggtctggtgctgattacttcagccaaaaggaacacctgtatatgTATGGACAGCAAGCGAACCG and gattacacttgttacccacataccacgaatcaggttatgcctcaTCAGAAGAACTCGTCAAGAAG (sequence in capital letters is of Tn *5*), a DNA fragment containing the kanamycin-resistance gene was synthesized. This fragment was electroporated into EDL933 containing pKM208, a thermosensitive plasmid bearing the bacteriophage lambda *exo* and *bet* genes (Murphy and Camellone (2003) ^15^) followed by selection for kanamycin-resistant clones. These were tested further by the ability of the cells to produce infective centers on strain AB1157 (Dewitt and Adelberg (1962) ^10^) and one of these was designated GM9251.

Prophage induction was accomplished by plating 10 µl of a standing overnight culture on solid medium and irradiating with 25 J/m ^2^ ultraviolet light followed by an overlay of 3.5 ml soft agar (0.3%) containing 300 µl of a standing overnight culture of the desired strain for bacteriophage propagation. Alternatively, the desired strain was mixed with dilutions of bacteriophage lysate at 37°C, incubated for 30 min, and incorporated in a soft agar layer on solid medium. In both induction and bacteriophage propagation, the plates were incubated at 37°C overnight. The next day, the soft agar layer was scraped off, and 4 ml of L broth or TM buffer (10 mM Tris-HCl, pH7.4, 10 mM MgSO _4_) was added together with 0.15 ml chloroform. The mixture was vortexed and left at room temperature for 10 min followed by centrifugation in a microfuge at room temperature for 2 min at 10,000 rpm. The supernatant was either used immediately or mixed with an equal volume of 4 M ammonium sulfate. The bacteriophage suspension in ammonium sulfate is stable for at least one month at 4°C in the dark. During lysate preparation it is crucial that the temperature remain at or above 20°C. Lysates were titered on AB1157.

For transduction, the recipient strain was grown standing overnight in liquid medium at 37°C, centrifuged, and resuspended in fresh medium. The recipient strain (100 µl) was mixed with varying amounts of bacteriophage lysate and 400 µl fresh medium. For selection using auxotrophic markers, the mixture was spread on selective minimal medium without further incubation. For drug-resistance markers, the mixture was incubated at 37°C for 60 min before plating on antibiotic medium. The plates were incubated at 37°C.

A 933W lysogenic derivative of *E. coli* K-12 was constructed as follows. Dilutions of a GM9251 induced lysate were mixed with a standing overnight L broth culture of strain MM294 and incubated at 37ºC for 60 min and portions added to L broth-kanamycin plates and incubating them overnight at 37ºC. Colonies were picked into L broth supplemented with kanamycin and grown overnight at 37ºC. The broth cultures were diluted and 10 µl portions placed onto a soft agar (0.3%) layer containing 200 µl of a standing overnight culture of AB1157 on an L broth plate. After allowing the drops to dry the plate was incubated overnight at 37ºC. A kanamycin-resistant lysogen that formed infective centers by this method was designated GM9255 and 933W phage induced from this lysogen was used in all experiments with *E. coli* K-12.

## Results and discussion

Initial experiments with bacteriophage 933W *stx2AB*::Kan were confounded by its instability. It was found that the bacteriophage was cold-sensitive and would not form plaques on indicator strains at 30°C but could do so at 37°C. The lysates rapidly lost infective titer (>90%) upon incubation at 4°C overnight. The addition of 2 M ammonium sulfate to the lysates stabilized the titer.

The observation that the bacteriophage 933W chromosome had terminal redundancy suggested that, like bacteriophage P1, it should be capable of transducing genetic markers. Strain KM80, which is derived from EDL933 and is not lysogenic for 933W, was mutagenized with ethyl methane sulfonate and the lactose, arabinose and maltose non-fermenting derivatives were isolated. These derivatives were used as recipients for transduction and, after mixing with bacteriophage from strain GM9251, were plated on minimal medium containing the appropriate carbon source. The results in Table 1 show that colonies were present on the plates with the mixture but not on plates with the recipient alone or the bacteriophage alone. Forty recombinants from each transduction were patched onto solid medium with kanamycin and all were sensitive.

**Table 1.** Transduction in enterohemorrhagic *Escherichia coli*.

**Selection**	**Transductants per 10 µl lysate**			
	**Plate 1**	**Plate 2**	**Plate 3**	**Average**
Lac ^+^	130	128	134	131
Ara ^+^	72	71	73	73
Mal ^+^	89	88	88	88

Lac ^-^ (GM9289), Ara ^-^ (GM9290) and Mal ^-^ (GM9291) derivatives of KM80 were transduced with a lysate prepared on GM9251. The number of transdcutants on each of three plates is shown followed by the average. There were no colonies on plates seeded with the recipient strain alone or from the GM9251 lysate.

A 933W *stx2AB*::Kan lysogen (GM9255) of *E. coli* K-12 strain MM294 was constructed and a lysate prepared on *E. coli* K-12 strain GM1748 ( *leu*::Tn *10*). This lysate was used to transduce strain AB1157 (Thr ^-^ Ara ^-^) to tetracycline-resistance (due to the Tn *10*). Of the 40 recombinants tested, 100% were Ara ^+^ and 100% Thr ^-^. Given that the gene order is *thr*- *ara*- *leu* and that the intervals between *thr* and *leu*, and *ara* and *leu*, are 80 and 14 kB respectively, the result indicates that cotransduction is possible for markers separated by 14 kB, but not 80 kB. This result is consistent with the 933W bacteriophage genome size of 61 kB. None of the 40 tetracycline-resistant recombinants were kanamycin-resistant, consistent with the transducing particles containing only host DNA.

Bacteriophage 933W is a member of a family of *stx*-encoding bacteriophages and these may also be capable of transduction. The annotation of a terminase in the 933W genome should be revised to a pacase to more clearly reflect the action of these enzymes. The ability of bacteriophage 933W to transduce genetic markers in EHEC and *E. coli* K-12 suggests that other related bacteria such as enteropathogenic *E. coli* or *Citrobacter rhodentium* may also be amenable to this type of genetic exchange. Only 933W non-lysogens have been used in the present study as recipients and it is not yet known if 933W lysogens can be transduced.

## Conclusion

Bacteriophage 933W is capable of transducing genetic markers in EHEC and *E. coli* K-12.
