# Supplementary material for: High efficiency generalized transduction in Escherichia coli O157:H7
Source: F1000Res. 2013 Jan 10;2:7. [Version 1] doi: 10.12688/f1000research.2-7.v1 (PMC3752737; doi:10.12688/f1000research.2-7.v1)
Supplement: Peer review history

# Peer review: Referee response for version 1

**Published:** 2013-01-21

**Authors:** Michael Donnenberg

**Affiliations:** School of Medicine, University of Maryland, Baltimore, MD, USA

The findings are novel and of potential interest in that generalized transduction, if confirmed in other pathogenic *E. coli* strains, could facilitate more rapid progress in understanding these infections. Such confirmation is necessary before its full impact can be appreciated.

I have read this submission. I believe that I have an appropriate level of expertise to confirm that it is of an acceptable scientific standard.

# Peer review: Referee response for version 1

**Published:** 2013-01-16

**Authors:** Donald Court

**Affiliations:** Gene Regulation and Chromosome Biology Laboratory, Center for Cancer Research, National Cancer Institute, Frederick, MD, USA

This paper describes methods used for *E. coli* O157:H7 (EHEC) to prepare phage lysates and to use those lysates for generalized transduction of bacterial markers, which is useful since P1 transduction in this strain requires the presence of a special gal mutant in any EHEC recipient. The general phage methods described are also useful. I have a few minor comments below to improve the paper.

- The authors point out that the kanR phage marker does not come along during the transduction event. See the last sentence of the abstract and the last sentence in the second paragraph of results. Both should say something to the effect that this indicates phage DNA is not involved instead of arguing that host DNA is. This is pretty obvious but I think the question is more whether there is some kind of host DNA pick into a phage genome.- It would be informative if the authors can provide a frequency of generalized transductants per phage particle (as well as per 10 microliters). What is the frequency of lysogeniziation  by this phage as measured by KanR? How does this transduction compare to a similar phage like P22.- How frequently does the tet marker transduce the EHEC strain?

I mark this as approved for publication with some minor revisions as suggested.

I have read this submission. I believe that I have an appropriate level of expertise to confirm that it is of an acceptable scientific standard.

# Peer review: Referee response for version 1

**Published:** 2013-01-14

**Authors:** Joe Wade

**Affiliations:** Wadsworth Center, New York State Department of Health, Albany, NY, USA

The authors describe a new transduction system for *E. coli* O157:H7, a strain that cannot be transduced using P1. Such a transducing phage would be a useful tool. The authors make a fairly convincing case that they have indeed isolated a transducing phage. However, there are some important, missing controls, and the efficiency of co-transduction of linked genes needs to be discussed in greater detail.

Specific comments:

1. Given that the impact of the transducing phage is likely to be limited to O157:H7 strains (i.e. not K-12), it is important to demonstrate transduction in O157:H7 more convincingly. Specifically, the authors should identify the specific mutation in the recipient strain and then show that this mutation is lost in the transductants. This will rule out spontaneous mutations as the cause of the phenotype. Alternatively, and perhaps more easily, the authors could attempt to transduce an antibiotic resistance gene, as they did for K-12.

2. The authors show that co-transduction occurs in K-12 between genes 14 kb apart. The frequency of co-transduction is 100% for 40 colonies tested. This is higher than I would have expected. Assuming random acquisition of host DNA by the phage, you would expect 8 or 9 colonies, on average, in which the two genes did not co-transduce. The probability of getting 40 out of 40 co-transductants would be 3e ^-5^. This suggests that the phage does not package host DNA randomly. The authors should discuss the significance of this, especially in light of the utility of the phage for generalised transduction.

I have read this submission. I believe that I have an appropriate level of expertise to confirm that it is of an acceptable scientific standard, however I have significant reservations, as outlined above.

# Response

**Published:** 2013-05-07

**Authors:** Martin Marinus

**Affiliations:** Department of Biochemistry & Molecular Pharmacology , University of Massachusetts Medical School, USA

1. There were never any colonies on the control plates for the O157:H7 transductions for three different markers so we consider the possibility that the transductants are spontaneous revertants to be very low.

2. We agree with the reviewer on this point and it is very possible that the DNA is not packaged randomly.
